# Focused Ultrasound-Induced Neurogenesis Requires an Increase in Blood-Brain Barrier Permeability

**DOI:** 10.1371/journal.pone.0159892

**Published:** 2016-07-26

**Authors:** Skyler J. Mooney, Kairavi Shah, Sharon Yeung, Alison Burgess, Isabelle Aubert, Kullervo Hynynen

**Affiliations:** 1 Physical Sciences, Sunnybrook Research Institute, Toronto, Ontario, Canada; 2 Biological Sciences, Sunnybrook Research Institute, Toronto, Ontario, Canada; 3 Department of Laboratory Medicine and Pathobiology, University of Toronto, Toronto, Ontario, Canada; 4 Department of Medical Biophysics, University of Toronto, Toronto, Ontario, Canada; Hungarian Academy of Sciences, HUNGARY

## Abstract

Transcranial focused ultrasound technology used to transiently open the blood-brain barrier, is capable of stimulating hippocampal neurogenesis; however, it is not yet known what aspects of the treatment are necessary for enhanced neurogenesis to occur. The present study set out to determine whether the opening of blood-brain barrier, the specific pressure amplitudes of focused ultrasound, and/or the intravenous administration of microbubbles (phospholipid microspheres) are necessary for the enhancement of neurogenesis. Specifically, mice were exposed to burst (10ms, 1Hz burst repetition frequency) focused ultrasound at the frequency of 1.68MHz and with 0.39, 0.78, 1.56 and 3.0MPa pressure amplitudes. These treatments were also conducted with or without microbubbles, at 0.39 + 0.78MPa or 1.56 + 3.0MPa, respectively. Only focused ultrasound at the ~0.78 MPa pressure amplitude with microbubbles promoted hippocampal neurogenesis and was associated with an increase in blood-brain barrier permeability. These results suggest that focused ultrasound -mediated neurogenesis is dependent upon the opening of the blood-brain barrier.

## Introduction

The blood-brain barrier (BBB) protects the brain and spinal cord from the entry of foreign compounds into the brain. However, it also prevents the access of >90% of the current pharmaceuticals, making brain diseases very difficult to treat. The ability to non-surgically increase the permeability of the BBB in a localized and controlled manner will likely drive innovation in centrally targeted pharmacology. Magnetic resonance (MR) imaging-guided focused ultrasound (FUS) is a technique with a demonstrated capability to transiently open the BBB in targeted regions of the brain [1]. When applied in conjunction with an intravenous injection of clinically approved microbubble contrast agents, FUS can produce BBB opening that is both focal and reversible [2]. Already, it has been demonstrated that this technology can be used to deliver pharmaceutical agents into the brain that are not normally able to cross the BBB in therapeutic amounts [3–5].

While transport of peripherally delivered substances into the brain is important for treating brain diseases, the exchange of endogenous vascular substances may also induce potentially beneficial effects in certain circumstances. For example, in a mouse model of Alzheimer’s disease (AD), FUS-mediated BBB opening permits increased levels of endogenous immunoglobulins to enter the brain, an event that may contribute to more effective clearance of β-amyloid [6]. In the same mouse model, it has been demonstrated that FUS-mediated BBB opening is capable of increasing hippocampal neurogenesis [7–8]. To date, it remains unknown whether the actual opening of the BBB is necessary for these neurogenic effects. FUS has been shown to selectively modulate the excitability of neural tissue in specific brain regions without accompanying BBB opening [9]. Furthermore, FUS without microbubbles, is capable of stimulating neurons and potentially increasing the density of brain-derived neurotrophic factor (BDNF) positive puncta in the CA1 and CA3 regions of the hippocampus [10]. Both neural stimulation and increased levels of BNDF are known to contribute to neurogenesis [11–12]. Therefore, the goal of this study is to determine what aspects of transcranial FUS are necessary for generating enhanced neurogenesis.

Previous studies from our lab used a protocol that was predefined with specific pressure amplitudes and that required the presence of microbubbles [7–8]. This protocol was developed to reliably and reversibly open the BBB. The present study tested this FUS protocol alongside others using different pressure amplitudes, in presence or absence of microbubbles, and with and without inducing BBB opening, in order to better understand what aspects of MR-guided FUS are crucial for stimulating the generation of new neurons.

## Methods

### Animals

16 adult C57BL/6 mice (20–66g) were used for this study. Animals were housed in the Sunnybrook Research Institute animal facility and had access to food and water ad libitum. Mice were assigned to groups described in Table 1. MR image-guided FUS was applied to the unilateral hippocampus at either 1.56MPa with microbubbles (group 1; n = 3), 0.39MPa with microbubbles (group 2; n = 3), 1.56MPa without microbubbles (group 3; n = 3) or 3.0MPa without microbubbles (group 4; n = 7). No animals demonstrated any symptoms requiring medical treatment or euthanasia due to FUS treatments. No animals became ill or required euthanasia prior to the experimental endpoint. All procedures were approved by the institutional Animal Care Committee (Sunnybrook Research institute, Toronto, Ontario, Canada) and were in accordance with guidelines provided by the Canadian Council on Animal Care and the Animals for Research Act.

**Table 1:** Specifications of focused ultrasound treatments for each group.

	Max. Pressure	Pressure Drop	Microbubbles
**Group 1**	1.56MPa (average)	50%	Yes
**Group 2**	0.39MPa	0%	Yes
**Group 3**	1.56MPa	0%	No
**Group 4**	3.00MPa	0%	No

### MR image-guided FUS

FUS was achieved using a custom-built transducer (1.68MHz) with a 75mm diameter and 60mm radius of curvature. The transducer was fitted with a custom-built polyvinylidene difluoride hydrophone in the centre. Under anaesthesia with isoflurane (2% @ 1L/min with medical air), the head of each animal was depilated. Each animal was then fitted with a tail vein catheter and laid in a supine position atop a sled that allowed coupling between the animal’s head and a degassed water bath. In order to register ultrasound focus parameters, T1- and T2- weighted scans were taken with a 7-Telsa MRI scanner (BioSpin 7030; Bruker, Billerica, Massachusetts). Immediately prior to sonication start, animals in groups 1 and 2 were administered the microbubble contrast agent Definity (Lantheus Medical Imaging, North Billerica, Massachusetts) intravenously at a dose of 0.02ml/kg (Table 1). FUS was then delivered unilaterally to a region containing the hippocampus in 10ms bursts at a 1Hz burst repetition frequency for 120s. For group 1, peak pressure was determined by the presence of ultraharmonic acoustic emissions, indicative of enhanced microbubble activity [13]. The average peak pressure for this group of animals was 1.56MPa. Once peak pressures were reached, the acoustic pressure was reduced to 50% of this maximum pressure for the remainder of the sonication [13]. For groups 2, 3 and 4, the acoustic pressure was set to ~25%(0.39MPa), ~100%(1.56MPa) and ~200%(3.00MPa) of the average peak pressure (Table 1). During the sonication, animals were administered the gadolinium-based contrast agent Gadovist (Schering AG, Berlin, Germany) intravenously at a dose of 0.2ml/kg in order to determine whether BBB opening had occurred. Contrast-enhanced T1-weighted images were then obtained for each animal.

### Immunohistological techniques

Beginning 42h following FUS treatments, animals were injected intraperitoneally with 5-Bromo-2′-deoxyuridine (BrdU) at a dose of 50mg/kg once a day for 6 days. Previous work from this lab used 4 days of BrdU treatment starting at 24 hours post-treatment [8]. Studies have shown that the integrity of the BBB opening is completely restored by 24 hours with very low or no MR contrast agents detectable in the brain after that timepoint [14]. In this study, we chose to start BrdU injections well after the 24 hour timeframe in order to ensure that the BBB had completely closed before BrdU injections began. Furthermore, we increased the number of administration days in order to try to capture a greater time period when neurogenesis might be stimulated after treatment. Animals survived for 12 days post BrdU treatment. Mice were deeply anaesthetized with a mixture of ketamine and xylazine and sacrificed by means of intracardiac perfusion with saline (0.9%) and 4% paraformaldehyde. Brains were removed, postfixed in paraformaldehyde and immersed in a 30% sucrose solution. Brain tissue was sliced on the coronal plane in 50μm sections on a cryostat and stored in cryoprotectant solution until staining. Every 6^th^ section of tissue was used for staining protocols. Tissue was first rinsed in a phosphate-buffered saline solution (PBS) and then incubated at room temperature in a blocking solution with 10% normal donkey serum for 1h. This was followed by incubation at 4°C in a solution containing goat anti-DCX (Santa Cruz Biotechnology, sc-8066) in a 1:200 dilution for 72h. Tissue was then rinsed in PBS and incubated at room temperature in a solution containing donkey anti-goat Alexa Fluor® 488 in a 1:200 dilution for 2h. Following a rinse in PBS, tissue was subjected to antigen retrieval in 2M HCl at 37°C for 35min, which was neutralized with 0.1M Borate buffer for 60s. After a further rinse in PBS, tissue was incubated overnight at 4°C in a solution containing rat anti-BrdU (Serotec #OBT0030) in a 1:400 dilution. Tissue was then rinsed in PBS and incubated at room temperature for 1h in a solution containing donkey anti-rat Cy3 in a 1:200 dilution. Tissue was then rinsed in PBS and incubated overnight at room temperature in a solution containing mouse anti-NeuN-biotin (Chemicon, #MAB377B) in a 1:200 dilution. Following a rinse in PBS, tissue was then incubated overnight at 4°C in a solution containing streptavidin Cy5 in a 1:200 dilution. Tissue was then rinsed in PBS and mounted on slides using VECTASHIELD® HardSet™ Mounting Medium (Vector Laboratories, Burlingame, CA). Because the treated hemisphere was not determinable in the histology slides for 4 animals in group 4, data will only be presented for the remaining 3 animals.

### Data collection and analysis

Analysis of the tissue was carried out by 2 different authors (S.Y. and S.J.M.). Z-stack images of the dentate gyrus were obtained with an inverted Zeiss Observer.Z1 microscope equipped with a Yokogawa CSU-X1 spinning disc confocal scanhead (Zeiss, Jena, Germany) using ZEN 2 software (blue edition; Zeiss, Jena, Germany). Groups 1–3 were imaged at 20X magnification and group 4 was imaged at 63X magnification. The number of total BrdU labelled cells, BrdU-DCX colocalized cells and BrdU-NeuN colocalized cells in a 1-in-6 series were counted using ZEN 2 or Stereo Investigator software (MBF Bioscience, Williston, Vermont). For tissue counted with Stereo Investigator, dissectors were 30μm in height with guard zones set to 5μm. 100% of the region of interest (dentate gyrus) was sampled in each tissue slice, effectively counting all cells in each hemisphere. These numbers were converted to an average score per tissue slice. For tissue counted using ZEN 2 software, all cells within the region of interest were marked and counted. These numbers, too, were converted to an average score per tissue slice. Because the untreated hemisphere served as the animal’s control, paired t-tests (two-tailed) were conducted using the average number of cells per tissue slice for each animal as the dependant variable and with the sonicated and control hemispheres as paired groups. This was done for BrdU labelled cells, BrdU-DCX colocalized cells and BrdU-NeuN colocalized cells.

## Results

### BBB opening

**Fig 1:**
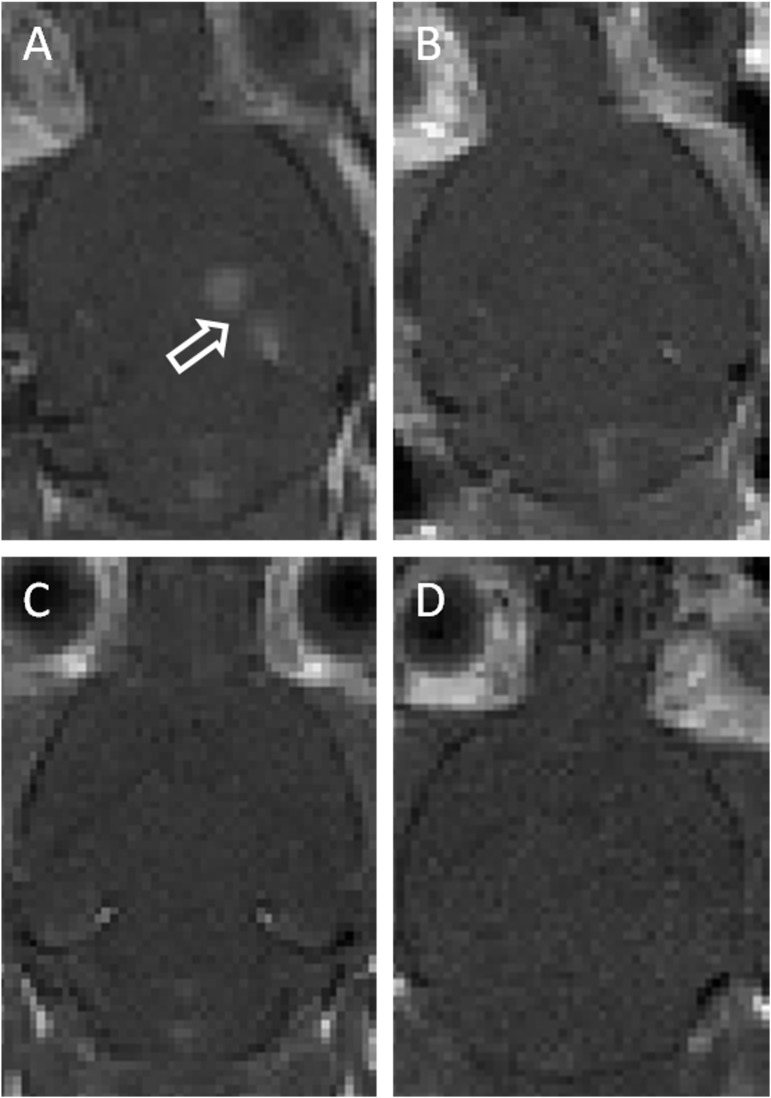
Representative post-sonication T1-weighted MR images of animals in (A) group 1 (~0.78 MPa pressure amplitudes with microbubbles), (B) group 2 (0.39MPa pressure amplitudes with microbubbles), (C) group 3 (1.56MPa pressure amplitudes without microbubbles), and (D) group 4 (3.0MPa pressure amplitudes without microbubbles). Only animals that received ~0.78MPa pressure amplitudes and microbubbles showed significant opening of the blood-brain barrier. Arrow indicates area of BBB opening.

Opening of the BBB was assessed by using the amount of gadolium-based contrast enhancement in post-treatment T1-weighted images. These images showed good BBB opening in the hippocampal region of animals in group 1 but no contrast enhancement was detected in groups 2–4 (Fig 1).

### Neurogenesis

**Fig 2:**
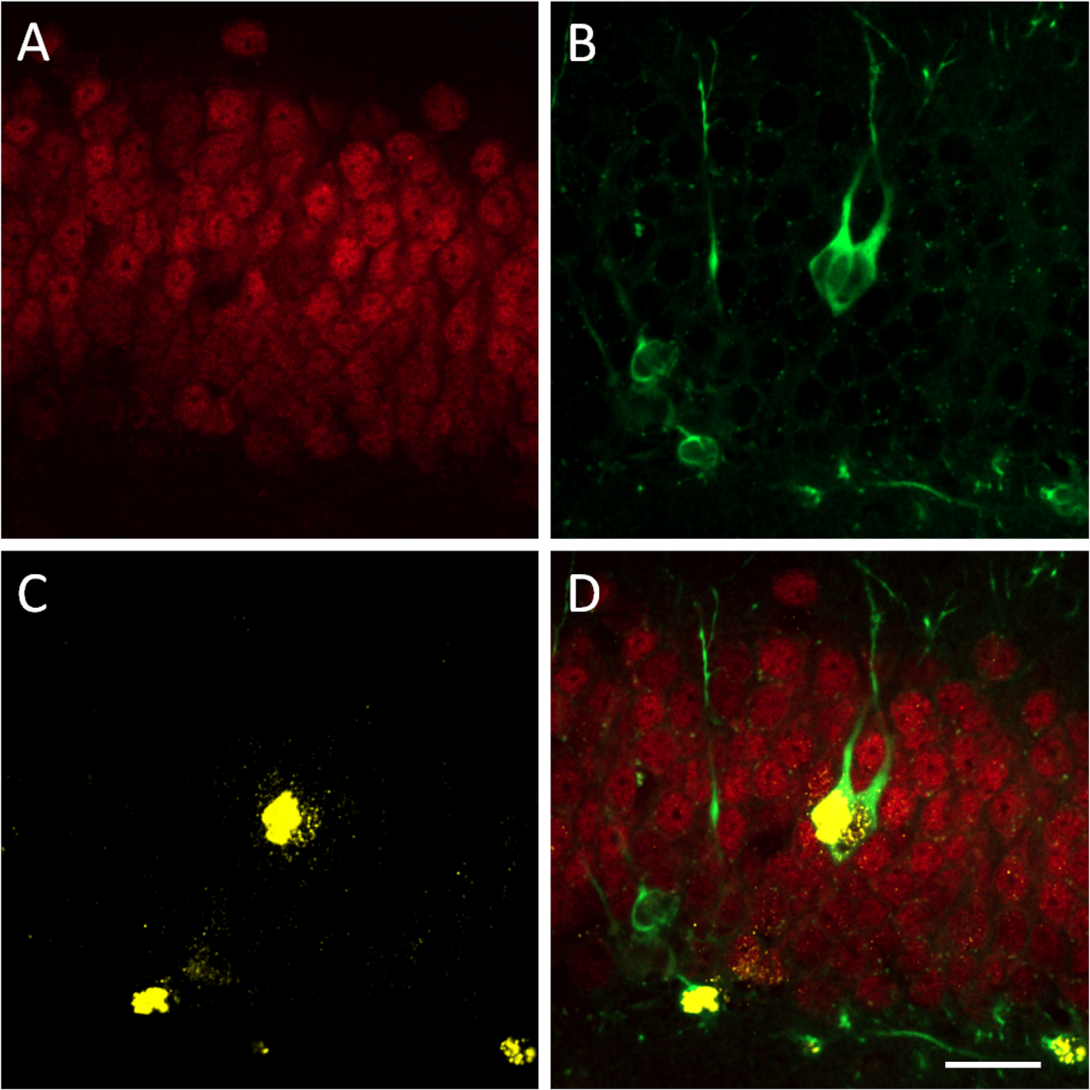
Confocal images of (A) NeuN, (B) DCX, (C) BrdU positive cells and (D) merged image of all cell-types (D) in the dentate gyrus 20 days after FUS treatment with daily injections with BrdU during days 2–7.

Staining for NeuN (Fig 2A) was prominent in the granular cell layer of the dentate gyrus, while DCX-labelled cell bodies were mostly confined to the subgranular layer with some migration into the granular layer (Fig 2B).

**Fig 3:**
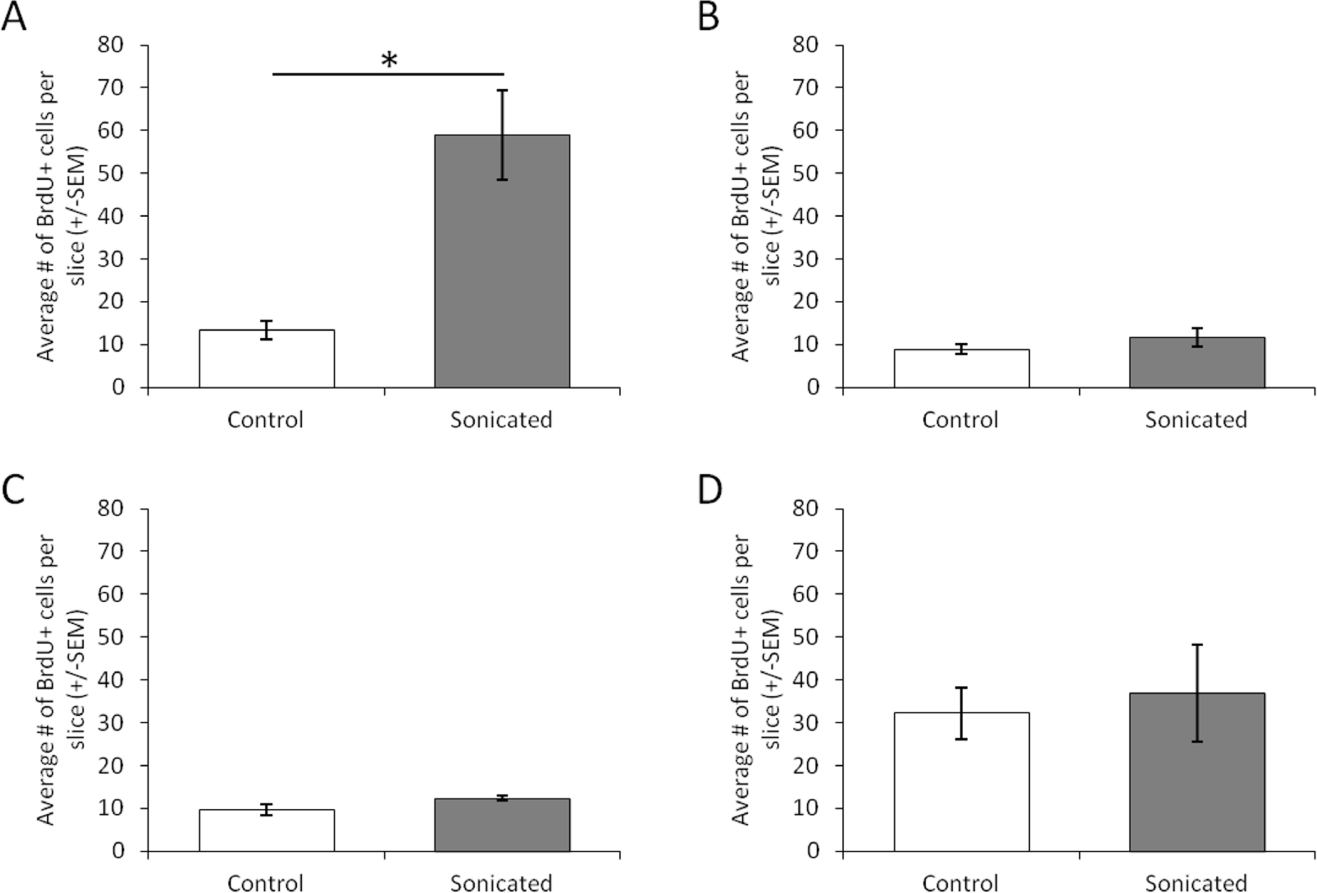
The average number of BrdU positive cells in each slice of tissue in the dentate gyrus of animals in (A) group 1 (~0.78 MPa pressure amplitudes with microbubbles), (B) group 2 (0.39MPa pressure amplitudes with microbubbles), (C) group 3 (1.56MPa pressure amplitudes without microbubbles), and (D) group 4 (3.0MPa pressure amplitudes without microbubbles). Error bars represent the standard error of the mean. Only animals that received ~0.78MPa pressure amplitudes and microbubbles showed significant increases in BRDU+ cells in the sonicated hemisphere. *p = 0.037

**Fig 4:**
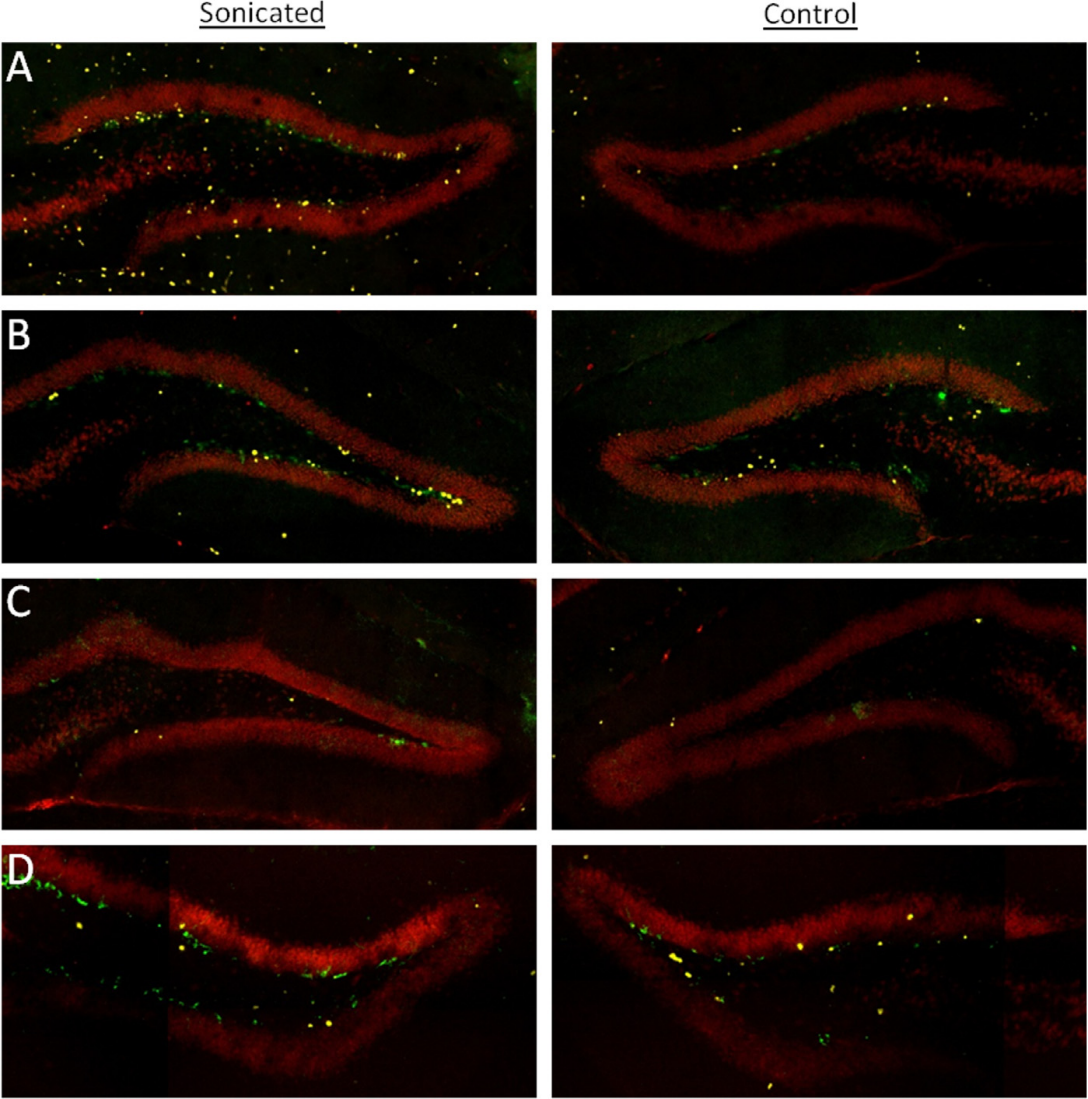
Confocal images of BrdU (yellow), NeuN (Red) and DCX (Green)-positive cells in the dentate gyrus of animals in (A) group 1 (~0.78 MPa pressure amplitudes with microbubbles), (B) group 2 (0.39MPa pressure amplitudes with microbubbles), (C) group 3 (1.56MPa pressure amplitudes without microbubbles), and (D) group 4 (3.0MPa pressure amplitudes without microbubbles).

**Fig 5:**
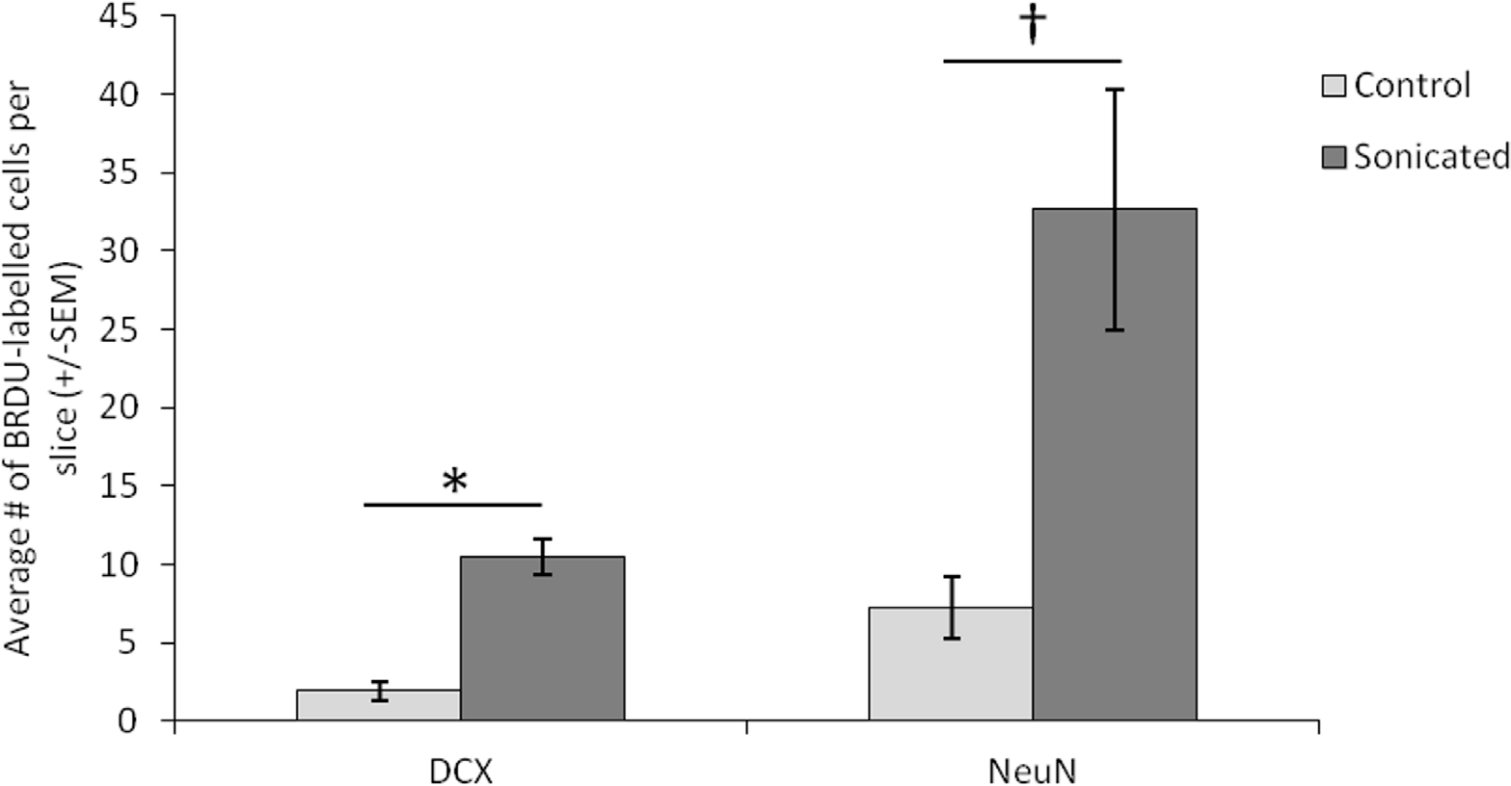
The average number of mature and immature neurons colabelled with BrdU in each slice of the dentate gyrus. Animals were from group 1 (~0.78 MPa pressure amplitudes with microbubbles). Error bars represent the standard error of the mean. † = 0.061, *p = 0.021.

FUS-mediated opening of the BBB (group 1) resulted in increased cell proliferation in the dentate gyrus of the sonicated hemisphere as measured by the average number of BrdU labelled cells in each slice of tissue (**Figs** 3A and 4A; *t*(3) = 5.022, *p* = 0.037). Due to the small number of animals, we conducted a poct-hoc power analysis in G*Power. This yielded a power level of 0.92, which is well above the recommended 0.80 level for detecting a modest effect size [15]. Importantly, this treatment increased the number of BRDU labelled cells that are colabelled with either DCX or NeuN indicating that this treatment induced neurogenesis specifically rather than just increasing general cell proliferation (*t*(3) = 4.512, *p* = 0.046). Post-hoc tests yielded a power level of 0.87 for this analysis. Delivering FUS at 0.39MPa with microbubbles (group 2), a procedure that did not open the BBB, did not produce this same increase in the average number of BrdU cells on the sonicated side (Figs 3B and 4B; *t*(3) = 1.377, *p* = 0.302). Similarly, animals receiving FUS without microbubbles failed to produce a hemispheric difference in the average numbers of BrdU labelled cells at either the at 1.56MPa (Figs 3C and 4C; *t*(3) = 2.596, *p* = 0.122) or 3.00MPa (Figs 3D and 4D; *t*(3) = 0.850, *p* = 0.485) pressure amplitudes. For neuron specific cell proliferation, FUS treatment which resulted in BBB opening was the only treatment to produce an increase in immature neurons colabelled with BrdU and DCX (Fig 5A; *t*(3) = 6.806, *p* = 0.021). Post-hoc analysis yielded a power level of 0.78. This was also the only treatment to produce an approaching significant increase in mature neurons that were colabelled with BrdU and NeuN (Fig 5A; *t*(3) = 3.870, *p* = 0.061). A post hoc power analysis conducted on G*Power revealed a power level of 0.54 and a needed n of 4 animals to reach the recommended power level of 0.80 [15]. FUS at 0.39MPa with microbubbles failed to produce an increase in mature (*t*(3) = 0.544, *p* = 0.641) or immature neurons (*t*(3) = 1.673, *p* = 0.236). Higher pressure amplitudes without microbubbles also failed to produce an increase in neurogenesis in the sonicated hemisphere. For animals receiving FUS at 1.56MPa, the two hemispheres showed no difference in the average numbers of BrdU-NeuN colocalized cells (*t*(3) = 1.523, *p* = 0.267) or BrdU-DCX colocalized cells (*t*(3) = 0.275, *p* = 0.809) in the dentate gyrus. Similarly, FUS at 3.0MPa produced no hemispheric difference in the average number of BrdU-NeuN colocalized cells (*t*(3) = 0.826, *p* = 0.496) or BrdU-DCX colocalized cells (*t*(3) = 0.832, *p* = 0.493) in the dentate gyrus.

## Discussion

The present study demonstrates that FUS-mediated BBB opening results in increased neurogenesis in accordance with previous studies [7–8]. In addition, we demonstrate that the same level of increased neurogenesis does not accompany sonication at lower pressure amplitudes with microbubbles or at higher pressure amplitudes without microbubbles, protocols which do not result in observable BBB opening. Previous work suggests FUS-induced BBB opening may also be accompanied by proliferation of other cell types, thereby maintaining the cellular environment [8].

A large amount of evidence suggests that blood vessels are an important aspect of neurogenic niches within the central nervous system ([16] for review). Important trophic factors in the circulation may contribute to the relationship between vascularisation and neurogenesis. For example, voluntary exercise increases the number of BrdU positive cells in the dentate gyrus which is dependent on the levels of serum insulin-like growth factor 1 [17]. In addition, circulating vascular endothelial growth factor (VEGF) is necessary for exercise‐induced adult hippocampal neurogenesis [18]. It has been shown that ultrasound treatment significantly up-regulates VEGF *in vitro* [19] and thus one possible explanation for the necessity of BBB opening during FUS treatment is the increased access to factors that are available in the vascular system. Another potential explanation is that the pressure amplitudes required for the stimulation to occur in the presence of microbubbles is so high that the BBB opening results as a by-product and is not required for the stimulation. Determining what vascular factors have the potential to contribute to increased neurogenesis promises to be a fruitful area of research going forward.

FUS has been shown to be both safe and reversible. The pressure amplitudes used here to stimulate neurogenesis resulted in very few extravasated erythrocytes and zero to few ischemic neurons or apoptotic cells in the sonicated regions of rabbits [2, 20]. Furthermore, these pressure amplitudes were not found to result in hemorrhaging in mice [21]. While treatment at these levels did result in an increase in microvacuolation and damage neurons at 30 minutes post treatment in mice, these effects had largely disappeared by 5 hours. In addition, the integrity of the BBB opening was completely restored by 24 hours after using this technique in rats [14]. These attributes suggest that the potential benefits of using this technique to induce neurogenesis outweigh the potential risks of exposing the brain parenchyma to the peripheral environment for extended periods of time.

The importance of understanding the dynamics of FUS-mediated neurogenesis lies in its clinical potential. Therapeutically increasing neurogenesis in the hippocampus is a promising treatment for mood disorders. A reduced hippocampal volume is a hallmark of depression patients [22–25]. Importantly, hippocampal neurogenesis is increased by most treatments that have antidepressant effects including pharmacologic agents, exercise, and electroconvulsive therapy [26–31]. With pharmacological interventions specifically, this increased neurogenesis is likely necessary for the treatment effects [30]. Furthermore, increasing neurogenesis to replace lost neurons has been proposed as a potential treatment for neurodegenerative disorders [32]. A recent study where FUS simultaneously increased the number of DCX cells in the dentate gyrus, increased dendritic branching, decreased plaque load and improved memory performance in a mouse model of AD supported this idea [7]. Therefore, neurogenesis and, in particular, FUS-mediated neurogenesis holds a great deal of promise for treating neurodegenerative disorders and other neural pathologies.

## Conclusion

The exact mechanisms through which FUS stimulates neurogenesis remain elusive. However, our data demonstrate that in the parameters tested, increased hippocampal neurogenesis occurs only when the BBB is transiently opened which supports the notion that it is a result of improved exchange with the components within the vascular environment. Neither microbubbles, nor increased pressure amplitudes appear to be capable of producing increased neurogenesis in the dentate gyrus of mice without accompanying BBB opening. Whether the opening of the BBB is a necessary component of treatment or a by-product of it, we have shown here that a specific treatment regime which causes BBB opening stimulates neurogenesis while others do not. A better understanding of the underlying biological mechanisms that are involved in the effects shown here may help move this technique to a point where it can be used to induce neurogenesis for therapeutic effects.

## Supporting Information

S1 File Experimental Dataset. (XLS)
